# The usability and reliability of a smartphone application for monitoring future dementia risk in ageing UK adults: CORRIGENDUM

**DOI:** 10.1192/bjp.2025.10369

**Published:** 2025-10

**Authors:** Graham Reid, Philip Vassilev, Jessica Irving, Triin Ojakäär, Liron Jacobson, Erin G. Lawrence, Jenny Barnett, Malika Tapparel, Ivan Koychev

In the original publication of the article, the total number of recruited participants for the study was mistakenly given as 756. This should have been 768.

The article has been updated to reflect this, and this corrigendum published.
